# Machine Learning for Prediction of Technical Results of Percutaneous Coronary Intervention for Chronic Total Occlusion

**DOI:** 10.3390/jcm12103354

**Published:** 2023-05-09

**Authors:** Tatsuya Nakachi, Masahisa Yamane, Koichi Kishi, Toshiya Muramatsu, Hisayuki Okada, Yuji Oikawa, Ryohei Yoshikawa, Tomohiro Kawasaki, Hiroyuki Tanaka, Osamu Katoh

**Affiliations:** 1 Department of Cardiology, Saiseikai Yokohamashi Nanbu Hospital, 3-2-10 Konandai, Konan-ku, Yokohama 234-0054, Japan; 2 Cardiology Department, Saitama Sekishinkai Hospital, 2-37-20 Irumagawa, Sayama, Saitama 350-1305, Japan; 3 Department of Cardiology, Tokushima Red Cross Hospital, 103 Irinokuchi, Komatsushima-cho, Komatsushima, Tokushima 773-8502, Japan; kkishi.trch@gmail.com; 4 Department of Cardiology, Tokyo Heart Center, 5-4-12 Kitashinagawa, Shinagawa-ku, Tokyo 141-0001, Japan; t-mura@tj8.so-net.ne.jp; 5 Department of Cardiology, Seirei Hamamatsu General Hospital, 2-12-12 Sumiyoshi, Naka-ku, Hamamatsu 430-8558, Japan; hisayuki@pat.hi-ho.ne.jp; 6 Department of Cardiovascular Medicine, The Cardiovascular Institute, 3-2-19 Nishiazabu, Minato-ku, Tokyo 106-0031, Japan; y-oikawa@cvi.or.jp; 7 Cardiology Center, Sanda City Hospital, 3-1-1 Keyakidai, Sanda, Hyogo 669-1321, Japan; ar147ttrs@gmail.com; 8 Department of Cardiology, Shin-Koga Hospital, 120 Tenjin-cho, Kurume, Fukuoka 830-8577, Japan; to-kawasaki@mug.biglobe.ne.jp; 9 Department of Cardiology, Kurashiki Central Hospital, 1-1-1 Miwa, Kurashiki, Okayama 710-8602, Japan; ht7307@kchnet.or.jp; 10 Department of Cardiology, Kusatsu Heart Center, 407-1 Komaizawa-cho, Kusatsu, Shiga 525-0014, Japan; okatoh653@mac.com

**Keywords:** chronic total coronary occlusion, percutaneous coronary intervention, machine learning

## Abstract

(1) Background: The probability of technical success in percutaneous coronary intervention (PCI) for chronic total occlusion (CTO) represents essential information for specifying the priority of PCI for treatment selection in patients with CTO. However, the predictabilities of existing scores based on conventional regression analysis remain modest, leaving room for improvements in model discrimination. Recently, machine learning (ML) techniques have emerged as highly effective methods for prediction and decision-making in various disciplines. We therefore investigated the predictability of ML models for technical results of CTO-PCI and compared their performances to the results from existing scores, including J-CTO, CL, and CASTLE scores. (2) Methods: This analysis used data from the Japanese CTO-PCI expert registry, which enrolled 8760 consecutive patients undergoing CTO-PCI. The performance of prediction models was assessed using the area under the receiver operating curve (ROC-AUC). (3) Results: Technical success was achieved in 7990 procedures, accounting for an overall success rate of 91.2%. The best ML model, extreme gradient boosting (XGBoost), outperformed the conventional prediction scores with ROC-AUC (XGBoost 0.760 [95% confidence interval {CI}: 0.740–0.780] vs. J-CTO 0.697 [95%CI: 0.675–0.719], CL 0.662 [95%CI: 0.639–0.684], CASTLE 0.659 [95%CI: 0.636–0.681]; *p* < 0.005 for all). The XGBoost model demonstrated acceptable concordance between the observed and predicted probabilities of CTO-PCI failure. Calcification was the leading predictor. (4) Conclusions: ML techniques provide accurate, specific information regarding the likelihood of success in CTO-PCI, which would help select the best treatment for individual patients with CTO.

## 1. Introduction

Despite progressive declines in cardiovascular mortality, coronary artery disease (CAD) remains the leading cause of death in developed countries [1]. For CAD, accumulated evidence has led to the standardization of treatment selection among percutaneous coronary intervention (PCI), coronary artery bypass grafting, or optimal medical treatment alone. However, due to the broad and heterogeneous spectrum of CAD patients, complex cases should be discussed individually to identify the optimal solution for each specific patient.

The probability of technical success for CTO-PCI represents essential information for specifying the priority of PCI in preprocedural discussions regarding treatment selection for CAD patients with CTO. Indeed, numerous scores for predicting CTO-PCI results have been derived based on regression analyses [2,3,4,5,6]. Nevertheless, the predictive ability of those scores remains modest at best [7,8], leaving room for improvements in model discrimination.

Machine learning (ML) techniques have emerged as highly effective methods for prediction and decision-making in a multitude of disciplines, including internet search engines, customized advertising, finance trending, and natural language processing [9,10]. When the goal is to generate a model that most accurately predicts an outcome, ML algorithms can prove quite advantageous over traditional regression methods. To date, the benefits of utilizing ML for predicting the technical results of CTO-PCI have not been evaluated on a large scale.

We therefore investigated the feasibility and accuracy of ML models for predicting the technical outcomes of CTO-PCI and compared their performances to the results from existing scores, including J-CTO [2], CL [4], and CASTLE [5] scores.

## 2. Materials and Methods

### 2.1. Study Population

This analysis used data from the Japanese CTO-PCI expert registry. This registry is a prospective, non-randomized study enrolling consecutive patients who are undergoing CTO-PCI performed by 46 highly experienced Japanese operators, all certified by the Japanese Board of CTO Interventional Specialists.

The requirements for certification are that the PCI operator has performed more than 300 CTO-PCIs and performs more than 50 CTO-PCIs per year. Certified specialists need to enrol all consecutive CTO-PCI datasets into the registry. The planned patient enrollment is from January 2014 to December 2022, and clinical follow-up will continue until December 2027. The design and enrollment status have been reported in detail [11,12]. Notably, an independent body of researchers (Clinical Research Center, Kurashiki Central Hospital, Ohara Healthcare Foundation, Okayama, Japan) monitors and controls data analysis, and procedural-related images (PCI angiograms, computed tomography images, and intravascular ultrasound images) are all uploaded into the central server of the core laboratory (Cardiovascular Imaging Center, Aichi, Japan) where independent physicians and technicians validate the content. This study protocol was approved by the review board of each institution, and written informed consent was obtained from all participants.

The study population was randomly divided into a training set (80%), from which ML models for predicting CTO-PCI results were derived, and a test set (20%), in which ML models and the existing scores were evaluated.

### 2.2. Definitions and Study Endpoint

Hyperlipidemia was defined as a total cholesterol level ≥220 mg/dL, a low-density lipoprotein cholesterol level ≥140 mg/dL, a high-density lipoprotein cholesterol level <40 mg/dL, a triglyceride level ≥150 mg/dL, or treatment for hyperlipidemia. Hemodialysis was defined as undergoing regular hemodialysis. The definition of CTO and the angiographic analysis of the target procedures have already been described [11,12]. The indication for CTO-PCI was completely left to the discretion of each operator and the discussion among the heart team of each institution. The selection of a CTO-PCI strategy depended on the operator’s discretion. The definitions of predictor variables for angiographic findings are provided in Supplemental Table S1. Viable CTO territory was defined as the presence of viability of myocardium in the perfusion territory of the target CTO lesion based on the findings of imaging modalities such as echocardiography, single photon emission computed tomography, cardiovascular magnetic resonance, or left ventriculography. Technical success was defined as successful guidewire CTO achieving <50% residual diameter stenosis without major side branch occlusion and thrombolysis in myocardial infarction flow grade 3. According to CTO-ARC consensus recommendations [13], in-hospital major adverse cardiovascular event included any of the following adverse events prior to hospital discharge: death, myocardial infarction, or clinically driven target vessel revascularization with PCI or coronary artery bypass grafting. Procedural success was defined as technical success plus the absence of an in-hospital major adverse cardiovascular event.

### 2.3. Predictor Variables

To ensure the availability of all predictor variables in prediction model development, we excluded variables with a missing data rate exceeding 20%. Missing values were filled with the median and mode of each continuous and categorical variable, respectively. To handle overfitting with regularization, continuous variables were normalized by z-scoring so that each continuous variable had both a mean of zero and a standard deviation of one. Multicategory variables were one-hot encoded in binary variables. Finally, a total of 65 predictor variables consisting of clinical and angiographic characteristics were used as independent predictor variables for model development.

### 2.4. ML Algorithm Models

To develop the prediction model for technical failure of CTO-PCI, we applied and compared the performances of 5 ML classifiers that are widely used in the literature: random forest; extreme gradient boosting (XGBoost); deep neural networks; support vector machine classifier; and L2-regularized logistic regression. For hyperparameter selection, a stratified 10-fold cross-validation and grid search was performed. The ranges of optimized hyperparameters for each classification algorithm are provided in Supplemental Table S2.

### 2.5. Comparison of Results from ML Models and Conventional Prediction Scores

We compared the performance of the developed ML algorithms with standard predictive multivariate logistic regression models: J-CTO, CL, and CASTLE scores. With the CASTLE score [5], the score component of “tortuosity” was defined as either 2 or more pre-occlusive bends of >90° or at least one bend of >120° in the CTO vessel. Because of the absence of identical findings obtained in the current registry, we used the finding “lesion bending”, defined as at least one bend of >45° throughout the occluded segment, as a substitute.

To compare ML models with those existing scores, we evaluated the existing scores directly on the test dataset, essentially performing an external validation of the prediction rules. However, comparing the external performance of those regression-based scores with the internal performance of ML algorithms could provide an unfair advantage to the ML algorithms. We therefore further developed a prediction score for technical failure of CTO-PCI in the training dataset using multivariate logistic regression analysis in a similar way with the existing scores. Potential predictive factors for CTO-PCI failure showing values of *p* < 0.005 in the univariate model were entered into the multivariate analysis. An integer scoring system (the CURRENT score) was developed by assigning points for each strong and independent predictor according to the beta coefficient and summing all points accrued. We also compared the predictive performance of ML models with that of the CURRENT score.

### 2.6. Evaluation Metrics

The models were evaluated in the test dataset, which was independent from the training dataset. Receiver operating characteristics (ROC) and precision/recall (PR) curve analysis were performed to assess the discriminatory ability of each ML model and the conventional prediction scores. Pairwise comparisons of the area under the ROC curves (ROC-AUC) were performed as described by Delong et al. [14].

Calibration of the best model (XGBoost) was evaluated using the Brier score method (range, 0–1) [15] and a figure comparing the observed and predicted risk of CTO-PCI failure.

### 2.7. Variable Importance

We also computed the variable importance of the best model (XGBoost) by measuring the average gain of splits using the variable across all decision trees within the model.

### 2.8. Software

Model development codes were developed in Python 3.6.6 (Python Software Foundation, Wilmington, DE, USA). The open-source library scikit-learn was used for the implementation of ML classifiers. The XGBoost 0.90 was used to build the XGBoost model.

### 2.9. Statistical Analysis

Data were statistically analyzed using SPSS Statistics version 24 (IBM, Armonk, NY, USA) and Medcalc version 20.110 statistical program (Medcalc, Ghent, Belgium). Continuous variables are presented as mean ± standard deviation. Categorical data are presented as frequencies and percentages. Normality was evaluated using the Shapiro–Wilk test. Normally distributed values were compared by unpaired *t*-test, and non-normally distributed values were compared by the Mann–Whitney U test. Categorical data were compared using the χ^2^ test or Fisher’s exact test.

We used logistic regression models for the training dataset to extract the score component of the CURRENT score by uni- and multivariate analyses. Given many variables, strong and independent predictor variables were identified using a stepwise approach with *p* < 0.005 as the inclusion criterion. All statistical tests were two-tailed and values of *p* < 0.05 were considered significant.

## 3. Results

### 3.1. Patient Characteristics

Among the 8760 CTO-PCI procedures performed between January 2014 and December 2019, technical success was achieved in 7990, representing an overall success rate of 91.2%. Each patient was randomly assigned to either the training cohort (80%, 7008 procedures) or the test cohort (20%, 1752 procedures). Patient characteristics in the training and test datasets are shown in Table 1 and Supplemental Table S3. Except for sex, smoking, hemodialysis, and viability of CTO territory, no significant differences in clinical or lesion-related characteristics were identified between the training and test cohorts.

Each training and test cohort was divided according to the technical outcome, and patient characteristics were analyzed (Supplemental Tables S4 and S5). In univariate analyses for the training dataset, patients with failed CTO-PCI were significantly more likely to have the following clinical characteristics: hypertension; diabetes; prior CABG; prior PCI; chronic occlusive pulmonary disease; arteriosclerosis obliterans; higher serum creatinine; and lower estimated glomerular filtration rate.

On uni- and multivariate logistic regression analysis for the training dataset, 8 variables were identified as strong independent predictors of the failure of CTO-PCI, collectively forming the CURRENT score (hemodialysis [+1], CTO vessel diameter < 2.5 mm [+1], CTO entry—no stump [+1], severe calcification [+2], lesion bending [+1], lesion length ≥ 20 mm [+1], proximal right coronary artery diseased [+1], and proximal left circumflex artery diseased [+1]) (Table 2).

### 3.2. Comparison of Prediction Models

**Figure 1:**
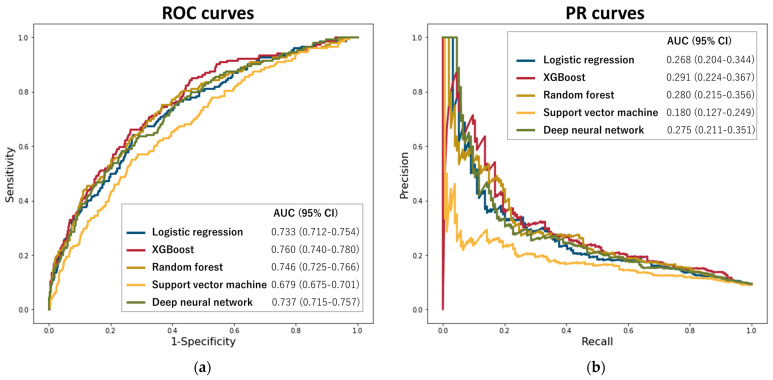
Areas under the ROC (**a**) and PR (**b**) curves for machine learning models to predict CTO-PCI failure. Abbreviations: PR, precision/recall; ROC, receiver operating characteristics; XGBoost, extreme gradient boosting.

Figure 1 shows the ROC and PR curves for the 5 ML models. Among these ML models, XGBoost exhibited the highest ROC-AUC (XGBoost: 0.760 [95% confidence interval {CI}: 0.740–0.780] vs. random forest: 0.746 [95%CI: 0.725–0.766], deep neural networks: 0.737 [95%CI: 0.715–0.757], L2-regularized logistic regression: 0.733 [95%CI: 0.712–0.754], and support vector machine classifier: 0.679 [95%CI: 0.657–0.701]) and PR-AUC (XGBoost: 0.291 [95%CI: 0.224–0.367] vs. random forest: 0.280 [95%CI: 0.215–0.356], deep neural networks: 0.275 [95%CI: 0.211–0.351], L2-regularized logistic regression: 0.268 [95%CI: 0.204–0.344], and support vector machine classifier: 0.180 [95%CI: 0.127–0.249]) for prediction of failed CTO-PCI. Regarding ROC-AUC, XGBoost allowed the prediction of CTO-PCI failure with higher accuracy than L2-regularized logistic regression (0.760 vs. 0.733, *p* = 0.034) and support vector machine classifier (0.760 vs. 0.679, *p* < 0.001). No significant differences were observed in predictive performance between XGBoost and random forest (0.760 vs. 0.746, *p* = 0.223) or deep neural networks (0.760 vs. 0.737, *p* = 0.071). In addition, XGBoost allowed an acceptable predictive ability for procedural failure (ROC-AUC 0.748 [95%CI: 0.727–0.768] and PR-AUC 0.323 [95%CI: 0.258–0.396]).

**Figure 2:**
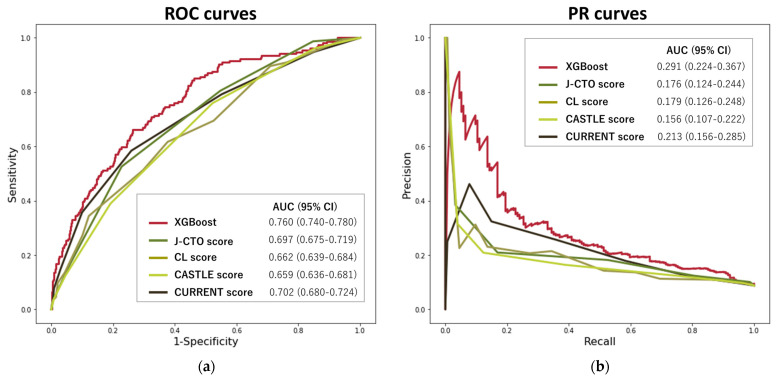
Areas under the ROC (**a**) and PR (**b**) curves comparing the best ML model with existing prediction scores for CTO-PCI failure. Abbreviations: CTO, chronic total occlusion; ML, machine learning; PCI, percutaneous coronary intervention; PR, precision/recall; ROC, receiver operating characteristics; XGBoost, extreme gradient boosting.

Figure 2 shows the ROC and PR curves for comparing the XGBoost model with the J-CTO, CL, CASTLE, and CURRENT scores. The prediction accuracy of the XGBoost model outperformed those of the conventional prediction scores: J-CTO (ROC-AUC 0.697 [95%CI: 0.675–0.719] and PR-AUC 0.176 [95%CI: 0.124–0.244]), CL (ROC-AUC 0.662 [95%CI: 0.639–0.684] and PR-AUC 0.179 [95%CI: 0.126–0.248]), CASTLE (ROC-AUC 0.659 [95%CI: 0.636–0.681] and PR-AUC 0.156 [95%CI: 0.107–0.222]), and CURRENT (ROC-AUC 0.702 [95%CI: 0.680–0.724] and PR-AUC 0.213 [95%CI: 0.156–0.285]). XGBoost exhibited a higher ROC-AUC than existing scores and the CURRENT score for prediction of failed CTO-PCI (*p* < 0.005 for all).

**Figure 3:**
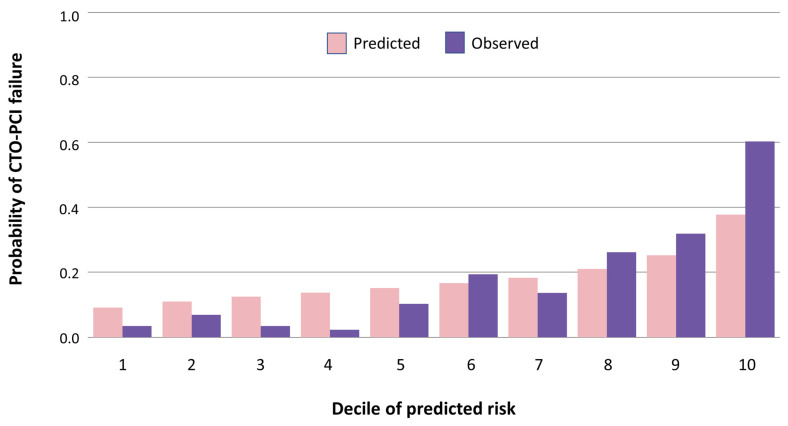
Observed versus predicted risk of CTO-PCI failure on XGBoost. Abbreviations: CTO, chronic total occlusion; PCI, percutaneous coronary intervention; XGBoost, extreme gradient boosting.

The Brier score for XGBoost was 0.074, indicating good calibration between the estimated predicted risk and observed risk of CTO-PCI failure. Calibration was also assessed by comparing estimated predicted and observed risk of CTO-PCI failure stratified by decile of predicted risk (Figure 3). A high correlation of predicted versus observed CTO-PCI failure was found (r = 0.97; *p* < 0.001).

### 3.3. Variable Importance

**Figure 4:**
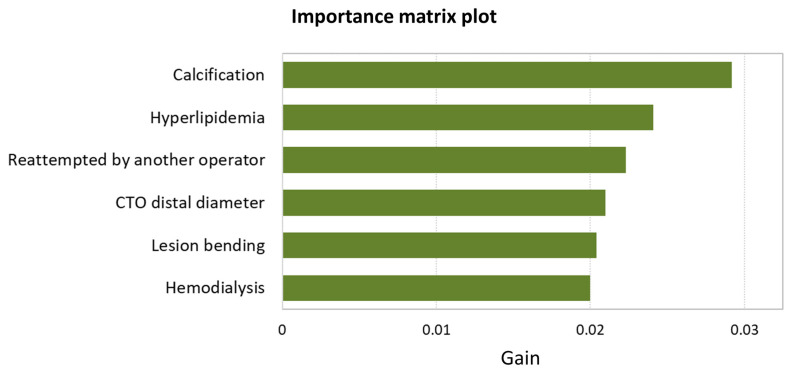
The importance matrix plot for XGBoost. Abbreviations: CABG, coronary artery bypass grafting; CTO, chronic total occlusion; XGBoost, extreme gradient boosting.

The importance matrix plot for XGBoost is shown in Figure 4. The first 6 variables contributing to the predictive performance of the XGBoost model were as follows: calcification; hyperlipidemia; reattempted by another operator; CTO distal diameter; lesion bending; and hemodialysis.

## 4. Discussion

This study had two major findings. First, XGBoost was our best-performing ML model for predicting CTO-PCI results. Second, XGBoost showed significantly better performance than existing scores for predicting the technical outcomes of CTO-PCI.

To the best of our knowledge, the present study represents the first large-scale, multicenter evaluation of ML for predicting the technical results of CTO-PCI. ML techniques provide accurate, specific information regarding the likelihood of success in CTO-PCI, which would optimize treatment selection for CAD patients with CTO in preprocedural discussions.

### 4.1. Prediction Accuracy of CTO-PCI Results

In the decision-making process for treatment selection when managing patients with complex CAD, recent revascularization guidelines have advocated a ‘Heart Team’ approach, referring to non-invasive cardiologists, anesthetists, and other specialists if deemed necessary [16]. A Heart Team approach facilitates more transparent decision-making but requires specific and accurate information regarding the likelihood of a successful result for each candidate’s procedural treatment instead.

To date, numerous risk prediction models for the results of CTO-PCI have been developed based on regression analysis, but the accuracy of those scores is modest at best [2,6]. Attempts to create new or additional scores have thus been made by integrating procedural algorithms [3] or increasing the number of patients included [5]. However, the predictive ability of scores has not been markedly improved through such efforts [7,8], emphasizing the need for improvements in model discrimination.

### 4.2. Advantages of ML Methods

ML models have been shown to work well when provided with large amounts of data [17,18,19,20], and the current registry provided data from 8760 procedures and 65 variables. Moreover, ML methods usually offer incremental gains in predictive performance while handling vast numbers of variable–variable interactions in each patient, effectively individualizing risk assessments and overcoming many limitations of standard statistical approaches using regression-based analysis [21]. In conventional prediction models for CTO-PCI results, most score components comprise angiographic findings of the CTO lesion and, in particular, the finding of severe calcification has been the most consistently included variable. However, the decision tree for CTO-PCI success in our previous report [22] showed that, among patients suffering CTO with severe calcification, no other angiographic findings affected CTO-PCI results. Additionally, a recent report showed apparent differences in the performance of prediction scores according to the procedural techniques applied, with higher predictability for patients who underwent CTO-PCI with antegrade-only procedures compared to those with bidirectional procedures [7]. Moreover, in the current study, hyperlipidemia was one of the leading predictors of CTO-PCI results. Hyperlipidemia has not been included as a score component among recently developed scoring systems based on regression analysis to gauge the likelihood of success in CTO-PCI. However, our previous report [11] showed that hyperlipidemia was an independent predictor of successful CTO-PCI in a primary retrograde approach, but not in overall or primary antegrade procedures. The effects of statin treatment on endothelium-mediated responses [23] and collateral development [24,25] of the coronary arteries might be beneficial for collateral channel crossing and retrograde procedures. Such findings suggest complex variable–variable interactions in clinical data for CTO-PCI, indicating incremental predictive performance by using ML methods, particularly for tree-based models.

### 4.3. Disadvantages of ML Methods

ML methods are usually more time-consuming than conventional regression analysis. Further, attention should be paid to the interpretation of the results of ML models. Important predictor variables may not be causal factors but just useful markers. The conversion to a points-based score based on coefficients for each variable obtained from conventional regression analysis cannot be applied to many ML methods.

To date, prediction models for CTO-PCI results have been developed to be as simple as possible, prioritizing the ease of remembering and calculation [2,3,4,5,6]. However, unlike those traditional scores from regression analysis, ML models require a computer for calculation and cannot be converted to a bedside arithmetical risk score. While the need to favor simplicity over accuracy might have been reasonable in the past, such considerations are no longer relevant within computerized medical care. Sufficient simplicity to be hand-calculable would not be acceptable if the trade-off were the sacrifice of accuracy in the prediction model providing critical information for treatment selection.

### 4.4. Future Directions

As described in the original report of the J-CTO score [2], which was originally developed to predict guidewire crossing within 30 min and remains the most widely applied score for technical results, clinical prediction systems should be continually updated to improve predictive performance by handling new data and optimizing algorithms. Recently, prediction models for CTO-PCI results, including coronary computed tomographic angiography (CCTA) findings, have shown relatively high performance [26,27]. CCTA offers advantages over CAG for direct visualization of CTO vessel trajectory, three-dimensional depiction of lesion bending, the distribution of calcification, and the presence of multiple occlusions. CCTA was not routinely obtained in the current registry, and ML prediction models for CTO-PCI results using CCTA findings have not yet been developed using a large-scale dataset. However, such analyses should be carried out in the future. Although experienced specialists have interpreted CCTA findings for developing prediction models, ML models such as neural networks might facilitate image interpretation and improve the predictive performance based on much larger datasets [28,29].

### 4.5. Limitations

This study has several potential limitations. First, the developed ML models have not been externally validated on a separate cohort. Second, in the CASTLE score [5], the score component of “tortuosity” was defined as either two or more pre-occlusive bends of >90° or at least one bend of >120° in the CTO vessel. Because of the absence of identical findings obtained in the current registry, we used the finding of “lesion bending”, defined as at least one bend of >45° throughout the occluded segment, as a substitute. This may have resulted in an underestimation of the performance of the CASTLE score. Finally, all CTO-PCIs were performed by highly experienced specialists, and the results may not be generalizable to the daily clinical practice of less-experienced operators. However, previous consensus reports on CTO have specified that CTO-PCI should be aggressively referred to a skilled operator [30]. As recent guidelines indicate, success rates for CTO-PCI are strongly associated with operator skillset, procedural volume, and the availability of dedicated equipment [16].

## 5. Conclusions

ML techniques improve the prediction of technical results of CTO-PCI. These techniques may help select the best treatment for individual patients with CTO in the standardized preprocedural discussion.

## Figures and Tables

**Table 1:** Patient characteristics.

	Overall Population	Training Set	Test Set	*p* Value
	(*n* = 8760)	(*n* = 7008)	(*n* = 1752)	
Age, years	67 ± 11	67 ± 11	67 ± 11	0.70
Female	1294 (14.8%)	1062 (15.2%)	232 (13.2%)	0.044
History of MI	4191 (47.8%)	3378 (48.2%)	813 (46.4%)	0.18
Prior CABG	615 (7.0%)	494 (7.0%)	121 (6.9%)	0.83
Hypertension	6801 (77.6%)	5451 (77.8%)	1350 (77.1%)	0.51
Diabetes mellitus	3954 (54.1%)	3173 (45.3%)	781 (44.6%)	0.60
Hyperlipidemia	7004 (80.0%)	5592 (79.8%)	1412 (80.6%)	0.46
Smoking				
Never	3481 (39.7%)	2830 (40.4%)	651 (37.2%)	0.046
Past	3821 (43.6%)	3021 (43.1%)	800 (45.7%)	
Current	1458 (16.6%)	1157 (16.5%)	301 (17.2%)	
ASO	1069 (12.2%)	834 (11.9%)	235 (13.4%)	0.084
Hemodialysis	585 (6.7%)	448 (6.4%)	137 (7.8%)	0.032
CCS				
0: The absence of anginal symptoms	4075 (46.5%)	3239 (46.2%)	836 (47.7%)	0.71
I	1794 (20.5%)	1438 (20.5%)	356 (20.3%)	
II	2463 (28.1%)	1992 (28.4%)	471 (26.9%)	
III	317 (3.6%)	250 (3.6%)	67 (3.8%)	
IV	111 (1.3%)	89 (1.3%)	22 (1.3%)	
Cr, mg/dL	1.23 ± 1.49	1.23 ± 1.49	1.23 ± 1.50	0.90
eGFR, mL/min/1.73 m^2^	63.4 ± 22.5	63.4 ± 22.5	63.5 ± 22.8	0.85
LVEF, %	54.2 ± 13.0	54.3 ± 13.0	53.9 ± 13.0	0.27
Viable CTO territory	8639 (98.6%)	6901 (98.5%)	1738 (99.2%)	0.020
Cerebrovascular disease	644 (7.4%)	512 (7.3%)	132 (7.5%)	0.74
EuroSCORE II	1.55 ± 2.23	1.57 ± 2.38	1.50 ± 1.44	0.26
Number of diseased vessels				
Single	3792 (43.3%)	3047 (43.5%)	745 (42.5%)	0.42
Double	2789 (31.8%)	2239 (31.9%)	550 (31.4%)	
Triple	2179 (24.9%)	1722 (24.9%)	457 (26.1%)	
CTO vessel				
Graft	3 (0.03%)	3 (0.04%)	0 (0%)	0.39
Right	4374 (49.9%)	3499 (49.9%)	875 (49.9%)	0.99
Left anterior descending	2814 (32.1%)	2267 (32.3%)	547 (31.2%)	0.37
Left circumferential	1535 (17.5%)	1210 (17.3%)	325 (18.6%)	0.21
Left main trunk	34 (0.4%)	29 (0.4%)	5 (0.3%)	0.44
In-stent occlusion	1165 (13.3%)	947 (13.5%)	218 (12.4%)	0.24
Blunt stump				
Tapered/tunnel	5720 (65.3%)	4548 (64.9%)	1172 (66.9%)	0.15
Blunt	1582 (18.1%)	1293 (18.5%)	289 (16.5%)	
No stump	1458 (16.6%)	1167 (16.7%)	291 (16.6%)	
Lesion calcification				
None	4342 (49.6%)	3482 (49.7%)	861 (49.1%)	0.95
Mild	2612 (29.8%)	2085 (29.8%)	527 (30.1%)	
Moderate	1187 (13.6%)	951 (13.6%)	236 (13.5%)	
Severe	618 (7.1%)	490 (7.0%)	128 (7.3%)	
Lesion bending	1848 (21.1%)	1480 (21.1%)	368 (21.0%)	0.92
Occlusion length				
<20 mm	3995 (45.1%)	3158 (45.1%)	797 (45.5%)	0.82
≥20 mm	4681 (53.4%)	3753 (53.6%)	928 (53.0%)	
Unmeasurable	124 (1.4%)	97 (1.4%)	27 (1.5%)	
Reattempted lesion				
No reattempt	7136 (81.5%)	5706 (81.4%)	1430 (81.6%)	0.85
Reattempted by the same operator	157 (1.8%)	124 (1.8%)	33 (1.9%)	0.75
Reattempted by another operator	1467 (16.7%)	1178 (16.8%)	289 (16.5%)	0.75
Collateral channel classification				
CC0	485 (5.5%)	387 (5.5%)	98 (5.6%)	0.99
CC1	3733 (42.6%)	2987 (42.6%)	746 (42.6%)	
CC2	4542 (51.8%)	3634 (51.9%)	908 (51.8%)	
Technical failure	770 (8.8%)	616 (8.8%)	154 (8.8%)	1.0
Procedural failure	872 (10.0%)	695 (9.9%)	177 (10.1%)	0.82
In-hospital MACE	134 (1.5%)	106 (1.5%)	28 (1.6%)	0.79
In-hospital death	24 (0.3%)	21 (0.3%)	3 (0.2%)	0.36
In-hospital MI	106 (1.2%)	84 (1.2%)	22 (1.3%)	0.85
Clinically driven TVR	10 (0.1%)	7 (0.1%)	3 (0.2%)	0.43

Values are presented as means ± standard deviation or as numbers (percentages). ASO, arteriosclerosis obliterans; CABG, coronary artery bypass grafting; CC, collateral channel; CCS, Canadian Cardiovascular Society; Cr, creatinine; CTO, chronic total occlusion; eGFR, estimated glomerular filtration rate; J-CTO, Multicenter CTO Registry in Japan; LVEF, left ventricular ejection fraction; MACE, major adverse cardiovascular event; MI, myocardial infarction; PCI, percutaneous coronary intervention; TVR, target vessel revascularization.

**Table 2:** Score components of the CURRENT score.

	Coefficient (β)	*p* Value	Odds Ratio (95% CI)	Score
Hemodialysis	0.695	<0.001	2.00 (1.49–2.70)	1
CTO vessel diameter < 2.5 mm	0.360	<0.001	1.43 (1.20–1.71)	1
CTO entry—no stump	0.422	<0.001	1.53 (1.26–1.84)	1
Severe calcification	0.897	<0.001	2.45 (1.94–3.10)	2
Lesion bending	0.545	<0.001	1.72 (1.45–2.06)	1
Length ≥ 20 mm	0.490	<0.001	1.63 (1.38–1.93)	1
Proximal RCA (AHA segment 1) diseased	0.284	<0.001	1.33 (1.23–1.56)	1
Proximal LCX (AHA segment 11) diseased	0.356	<0.001	1.43 (1.18–1.73)	1

AHA, American Heart Association; CABG, coronary artery bypass grafting; CI, confidence interval; CTO, chronic total occlusion; LCX, left circumflex artery; RCA, right coronary artery.

## Data Availability

The data presented in this study are available on request from the corresponding authors.

## Supplementary Materials

The following supporting information can be downloaded at: https://www.mdpi.com/article/10.3390/jcm12103354/s1; Table S1: Definitions of the predictor variables for angiographic findings; Table S2: The ranges of optimized hyperparameters for machine learning models; Table S3: Patient characteristic in the training and test cohort; Table S4: Patient characteristics in the training cohort; Table S5: Patient characteristics in the test cohort.

## References

[1] Tsao C.W., Aday A.W., Almarzooq Z.I., Alonso A., Beaton A.Z., Bittencourt M.S., Boehme A.K., Buxton A.E., Carson A.P., Commodore-Mensah Y. (2022). Heart Disease and Stroke Statistics—2022 Update: A Report from the American Heart Association. Circulation.

[2] Morino Y., Abe M., Morimoto T., Kimura T., Hayashi Y., Muramatsu T., Ochiai M., Noguchi Y., Kato K., Shibata Y. (2011). Predicting Successful Guidewire Crossing through Chronic Total Occlusion of Native Coronary Lesions within 30 Minutes: The J-CTO (Multicenter CTO Registry in Japan) Score as a Difficulty Grading and Time Assessment Tool. JACC Cardiovasc. Interv..

[3] Christopoulos G., Kandzari D.E., Yeh R.W., Jaffer F.A., Karmpaliotis D., Wyman M.R., Alaswad K., Lombardi W., Grantham J.A., Moses J. (2016). Development and validation of a novel scoring system for predicting technical success of chronic total occlusion percutaneous coronary interventions: The PROGRESS CTO (Prospective Global Registry for the Study of Chronic Total Occlusion Intervention) Score. JACC Cardiovasc. Interv..

[4] Alessandrino G., Chevalier B., Lefèvre T., Sanguineti F., Garot P., Unterseeh T., Hovasse T., Morice M.C., Louvard Y. (2015). A clinical and angiographic scoring system to predict the probability of successful first-attempt percutaneous coronary intervention in patients with total chronic coronary occlusion. JACC Cardiovasc. Interv..

[5] Szijgyarto Z., Rampat R., Werner G.S., Ho C., Reifart N., Lefevre T., Louvard Y., Avran A., Kambis M., Buettner H.J. (2019). Derivation and Validation of a Chronic Total Coronary Occlusion Intervention Procedural Success Score from the 20,000-Patient EuroCTO Registry: The EuroCTO (CASTLE) Score. JACC Cardiovasc. Interv..

[6] Galassi A.R., Boukhris M., Azzarelli S., Castaing M., Marzà F., Tomasello S.D. (2016). Percutaneous coronary revascularization for chronic total occlusions: A novel predictive score of technical failure using advanced technologies. JACC Cardiovasc. Interv..

[7] Karatasakis A., Danek B.A., Karmpaliotis D., Alaswad K., Jaffer F.A., Yeh R.W., Patel M., Bahadorani J.N., Lombardi W.L., Wyman R.M. (2016). Comparison of various scores for predicting success of chronic total occlusion percutaneous coronary intervention. Int. J. Cardiol..

[8] Kalogeropoulos A.S., Alsanjari O., Keeble T.R., Tang K.H., Konstantinou K., Katsikis A., Jagathesan R., Aggarwal R.K., Clesham G.J., Kelly P.A. (2020). CASTLE score versus J-CTO score for the prediction of technical success in chronic total occlusion percutaneous revascularisation. Eurointervention.

[9] Waljee A.K., Higgins P.D.R. (2010). Machine Learning in Medicine: A Primer for Physicians. Am. J. Gastroenterol..

[10] Deo R.C. (2015). Machine Learning in Medicine. Circulation.

[11] Suzuki Y., Tsuchikane E., Katoh O., Muramatsu T., Muto M., Kishi K., Hamazaki Y., Oikawa Y., Kawasaki T., Okamura A. (2017). Outcomes of Percutaneous Coronary Interventions for Chronic Total Occlusion Performed by Highly Experienced Japanese Specialists: The First Report from the Japanese CTO-PCI Expert Registry. JACC Cardiovasc. Interv..

[12] Tanaka H., Tsuchikane E., Muramatsu T., Kishi K., Muto M., Oikawa Y., Kawasaki T., Hamazaki Y., Fujita T., Katoh O. (2019). A Novel Algorithm for Treating Chronic Total Coronary Artery Occlusion. J. Am. Coll. Cardiol..

[13] Ybarra L.F., Rinfret S., Brilakis E.S., Karmpaliotis D., Azzalini L., Grantham J.A., Kandzari D.E., Mashayekhi K., Spratt J.C., Wijeysundera H.C. (2021). Definitions and Clinical Trial Design Principles for Coronary Artery Chronic Total Occlusion Therapies: CTO-ARC Consensus Recommendations. Circulation.

[14] Delong E.R., Delong D.M., Clarke-Pearson D.L. (1988). Comparing the Areas under Two or More Correlated Receiver Operating Characteristic Curves: A Nonparametric Approach. Biometrics.

[15] Brier G.W. (1950). Verification of forecasts expressed in terms of probability. Mon. Weather. Rev..

[16] Neumann F.J., Sousa-Uva M., Ahlsson A., Alfonso F., Banning A.P., Benedetto U., Byrne R.A., Collet J.P., Falk V., Head S.J. (2019). 2018 ESC/EACTS Guidelines on myocardial re-vascularization. Eur. Heart J..

[17] Rajkomar A., Dean J., Kohane I. (2019). Machine Learning in Medicine. N. Engl. J. Med..

[18] Westcott R.J., Tcheng J.E. (2019). Artificial Intelligence and Machine Learning in Cardiology. JACC Cardiovasc. Interv..

[19] Dorado-Díaz P.I., Sampedro-Gómez J., Vicente-Palacios V., Sánchez P.L. (2019). Applications of Artificial Intelligence in Cardiology. The Future is Already Here. Rev. Esp. Cardiol. (Engl. Ed.).

[20] Heckman G.A., Hirdes J.P., McKelvie R.S. (2020). The Role of Physicians in the Era of Big Data. Can. J. Cardiol..

[21] Goldstein B.A., Navar A.M., Carter R.E. (2017). Moving beyond regression techniques in cardiovascular risk prediction: Applying machine learning to address analytic challenges. Eur. Heart J..

[22] Nakachi T., Kohsaka S., Yamane M., Muramatsu T., Okamura A., Kashima Y., Matsuno S., Sakurada M., Kijima M., Tanabe M. (2017). Impact of Hemodialysis on Procedural Outcomes of Percutaneous Coronary Intervention for Chronic Total Occlusion: Insights from the Japanese Multicenter Registry. J. Am. Heart Assoc..

[23] Treasure C.B., Klein J.L., Weintraub W.S., Talley J.D., Stillabower M.E., Kosinski A.S., Zhang J., Boccuzzi S.J., Cedarholm J.C., Alexander R.W. (1995). Beneficial Effects of Cholesterol-Lowering Therapy on the Coronary Endothelium in Patients with Coronary Artery Disease. N. Engl. J. Med..

[24] Pourati I., Kimmelstiel C., Rand W., Karas R.H. (2003). Statin use is associated with enhanced collateralization of severely diseased coronary arteries. Am. Heart J..

[25] Liu L., Gao L., Tan H., Qi Y., Cui D., Wang Z., Liu J. (2022). Effect of different doses of atorvastatin on collateral formation in coronary artery disease patients with coronary atherosclerosis. Coron. Artery Dis..

[26] Yu C.W., Lee H.J., Suh J., Lee N.H., Park S.M., Park T.K., Yang J.H., Song Y.B., Hahn J.Y., Choi S.H. (2017). Coronary Computed Tomography Angiography Predicts Guidewire Crossing and Success of Percutaneous Intervention for Chronic Total Occlusion: Korean Multicenter CTO CT Registry Score as a Tool for Assessing Difficulty in Chronic Total Occlusion Percutaneous Coronary Intervention. Circ. Cardiovasc. Imaging.

[27] Fujino A., Otsuji S., Hasegawa K., Arita T., Takiuchi S., Fujii K., Yabuki M., Ibuki M., Nagayama S., Ishibuchi K. (2018). Accuracy of J-CTO Score Derived from Computed Tomography versus Angiography to Predict Successful Percutaneous Coronary Intervention. JACC Cardiovasc. Imaging.

[28] Tu J.V. (1996). Advantages and disadvantages of using artificial neural networks versus logistic regression for predicting medical outcomes. J. Clin. Epidemiol..

[29] Liu M.-H., Zhao C., Wang S., Jia H., Yu B. (2022). Artificial Intelligence—A Good Assistant to Multi-Modality Imaging in Managing Acute Coronary Syndrome. Front. Cardiovasc. Med..

[30] Thompson C.A., Jayne J.E., Robb J.F., Friedman B.J., Kaplan A.V., Hettleman B.D., Niles N.W., Lombardi W.L. (2009). Retrograde techniques and the impact of operator volume on percutaneous intervention for coronary chronic total occlusions an early U.S. experience. JACC Cardiovasc. Interv..

