# Supplementary material for: Machine Learning for Prediction of Technical Results of Percutaneous Coronary Intervention for Chronic Total Occlusion
Source: J Clin Med. 2023 May 9;12(10):3354. doi: 10.3390/jcm12103354 (PMC10218988; doi:10.3390/jcm12103354)
Supplement: Supplementary file 1 [file jcm-12-03354-s001.zip › suppleTableS1-JCM.pdf]

Supplemental Table S1. Definitions of the predictor variables for angiographic findings

Predictor variable		Definition
Collateral channel classification	CC0<br>CC1<br>CC2	The angiographic assessment of collateral connections was made according to the Werner classification.
CTO distal visibility	Good<br>Fair<br>Invisible	Opacification beyond the occlusion was classified into either none, faint, or good. Good distal visibility was defined as distal opacification comparable to the proximal segment.
CTO entry	Tapered/tunnel<br>Blunt<br>No stump	The angiographic morphology of the proximal cap was classified as tapered if the occluded segment ended proximally in a funnel-shaped form, blunt if it did not, and no stump if the guidewire target was not angiographically apparent.
Calcification	Non<br>Mild<br>Moderate<br>Severe	Lesion calcification was assigned to 1 of 4 categories: non, mild (spots), moderate (involving ≤50% of the reference lesion diameter), and severe (involving >50% of the reference lesion diameter).
Lesion bending		Lesion bending was defined as at least one bend of >45° assessed using angiography throughout the occluded segment.
Proximal tortuosity	Straight<br>Mild<br>Moderate<br>Severe	Tortuosity of the proximal segment to the CTO lesion was classified as follows. Mild tortuosity was defined as the presence of one bend >70° and <90°, moderate tortuosity was defined as a segment containing either 2 bends >70° or 1 bend >90°, and severe tortuosity was defined as the presence of two bends >90° or one bend >120°.
Lesion length	<20 mm<br>≥20 mm<br>Unmeasurable	The length of coronary occlusions was estimated from angiographic projections visually with single- or dual-contrast injections. Occlusion length was categorized as either <20 or ≥20 mm.

CC, collateral channel; CTO, chronic total occlusion.
