# Supplementary material for: Machine Learning for Prediction of Technical Results of Percutaneous Coronary Intervention for Chronic Total Occlusion
Source: J Clin Med. 2023 May 9;12(10):3354. doi: 10.3390/jcm12103354 (PMC10218988; doi:10.3390/jcm12103354)
Supplement: Supplementary file 1 [file jcm-12-03354-s001.zip › suppleTableS2-JCM.pdf]

Supplemental Table S2. The ranges of optimized hyperparameters for machine learning models

**Algorithms**	**Fixed hyperparameters**	**Ranges of tuned hyperparameters**
L2-regularized logistic regression	penalty = ‘l2’<br>max_iter = 1000	C: [0.001, 0.005,0.01, 0.05, 0.1, 0.5, 1]<br>solver: ['newton-cg', 'lbfgs', 'liblinear', 'sag', 'saga']
Random forest		max_features: [‘log2’, ‘auto’, ‘sqrt’]<br>min_samples_split: [2, 3, 5]<br>min_samples_leaf: [1, 3, 5]<br>bootstrap: [True,False]<br>n_estimators: [50, 100, 150]<br>criterion: [‘gini’, ‘entropy’]
XGBoost	n_estimators = 100	subsample: [0.6, 0.8, 1.0]<br>max_depth: [3,4,5]<br>learning_rate: [0.01, 0.05, 0.1]<br>min_child_weight: [1.0, 2.0, 3.0]<br>colsample_bytree: [0.6, 0.8,1.0]
Deep neural network		alpha: [0.1, 0.01, 0.02, 0.005, 0.0001,0.00001]<br>hidden_layer_sizes: [(10,10,10), (100,100,100), (100,100), (3,5), (5, 3)]<br>solver: [‘lbfgs’, ‘adam’, ‘sgd’]<br>activation: [‘relu’, ‘logistic’]

Support vector machine classifier	kernel = ‘rbf’	gamma: [0.001, 0.01, 0.1, 1]<br>C: [1,10,100]
Final model: XGBoost<br>XGBoost hyperparameters:<br>XGBClassifier (colsample_bytree = 0.6, learning_rate = 0.05, max_depth = 3, min_child_weight = 2.0, n_estimators = 100, subsample = 0.8)		

XGBoost, extreme gradient boosting.
