# Supplementary material for: Machine Learning for Prediction of Technical Results of Percutaneous Coronary Intervention for Chronic Total Occlusion
Source: J Clin Med. 2023 May 9;12(10):3354. doi: 10.3390/jcm12103354 (PMC10218988; doi:10.3390/jcm12103354)
Supplement: Supplementary file 1 [file jcm-12-03354-s001.zip › suppleTableS3-JCM.pdf]

Supplemental Table S3. Patient characteristic in the training and test cohort

		**Training cohort (n=7008)**	**Test cohort (n=1752)**	***P* value**
Age, years		67.4±11.1	67.3±10.8	0.70
Female		1062 (15.2%)	232 (13.2%)	0.044
Hypertension		5451 (77.8%)	1350 (77.1%)	0.51
Hyperlipidemia		5592 (79.8%)	1412 (80.6%)	0.45
Diabetes		3173 (45.3%)	781 (44.6%)	0.24
Smoking status	Never	2830 (40.4%)	651 (37.2%)	0.046
	Past	3021 (43.1%)	800 (45.7%)	
	Current	1157 (16.5%)	301 (17.2%)	
History of MI		3378 (48.2%)	8183 (46.4%)	0.18
Prior CABG		494 (7.0%)	121 (6.9%)	0.83
Prior PCI		4780 (68.2%)	1205 (68.8%)	0.65
Cerebrovascular disease		512 (7.3%)	132 (7.5%)	0.74
Cr, mg/dL		1.23±1.49	1.23±1.50	0.90
eGFR, mL/min/1.73 m^2		63.4±22.5	63.5±22.8	0.85
Hemodialysis		448 (6.4%)	137 (7.8%)	0.032
Chronic occlusive pulmonary disease		164 (2.3%)	50 (2.9%)	0.21
Arteriosclerosis obliterans		834 (11.9%)	235 (13.4%)	0.084
Malignancy		192 (2.7%)	53 (3.0%)	0.52
EuroSCORE II		1.57±2.38	1.50±1.44	0.26
LVEF, %		54.3±13.0	53.9±13.0	0.27
NYHA class	Not heart failure	5528 (78.9%)	1383 (78.9%)	0.86
	I	646 (9.2%)	155 (8.8%)	
	II	617 (8.8%)	165 (9.4%)	
	III	155 (2.2%)	36 (2.1%)	

	IV	62 (0.9%)	13 (0.7%)	
CCS class	Asymptomatic	3239 (46.2%)	836 (47.7%)	0.71
	I	1438 (20.5%)	356 (20.3%)	
	II	1992 (28.4%)	471 (26.9%)	
	III	250 (3.6%)	67 (3.8%)	
	IV	89 (1.3%)	22 (1.3%)	
ST-T wave abnormality on ECG		1688 (23.8%)	426 (24.3%)	0.65
Abnormal Q wave on ECG		1897 (27.1%)	461 (26.3%)	0.52
Stress Test	Not performed	6001 (85.6%)	1529 (87.3%)	0.077
	Negative study	333 (4.8%)	76 (4.3%)	0.46
	Positive study	632 (9.0%)	138 (7.9%)	0.13
	Equivocal study	42 (0.6%)	9 (0.5%)	0.67
Wall Motion in the perfusion territory of CTO	Normal	2780 (39.7%)	678 (38.7%)	0.39
	Hypokinesis	3896 (55.6%)	1003 (57.2%)	
	Akinesis	316 (4.5%)	66 (3.8%)	
	Dyskinesis	16 (0.2%)	5 (0.3%)	
Viable CTO territory		6901 (98.5%)	1738 (99.2%)	0.020
Diagnosis	Unstable angina pectoris	186 (2.7%)	52 (3.0%)	0.47
	Silent myocardial ischemia	2624 (37.4%)	681 (38.9%)	0.27
	Stable angina pectoris	3056 (43.6%)	759 (43.3%)	0.83
	Old myocardial infarction	1075 (15.3%)	243 (13.9%)	0.12
	Acute myocardial infarction	67 (1.0%)	17 (1.0%)	0.96
Other than the operator's affiliated institution		2359 (33.7%)	587 (33.5%)	0.90
Grafted CTO vessel	CTO vessel grafted and graft failure (-)	87 (1.2%)	26 (1.5%)	0.42
	CTO vessel grafted and graft failure (+)	311 (4.4%)	71 (4.1%)	0.48

	Not grafted CTO vessel	6610 (94.3%)	1655 (94.5%)	0.82
Initially planned strategy, Primary bidirectional approach		2045 (29.2%)	513 (29.3%)	0.93
Number of diseased vessels	Single	3047 (43.5%)	745 (42.5%)	0.42
	Double	2239 (31.9%)	550 (31.4%)	
	Triple	1722 (24.6%)	457 (26.1%)	
Target CTO vessel	RCA	3499 (49.9%)	875 (49.9%)	0.99
	LAD	2267 (32.3%)	547 (31.2%)	0.37
	LCX	1210 (17.3%)	325 (18.6%)	0.21
	LM	29 (0.4%)	5 (0.3%)	0.44
	Graft	3 (0.04%)	0 (0%)	0.39
CTO location	Distal	736 (10.5%)	201 (11.5%)	0.24
	Mid	2940 (42.0%)	717 (40.9%)	0.44
	Proximal	3050 (43.5%)	759 (43.3%)	0.88
	Ostium	282 (4.0%)	75 (4.3%)	0.63
Collateral channel classification	CC0	387 (5.5%)	98 (5.6%)	0.99
	CC1	2987 (42.6%)	746 (42.6%)	
	CC2	3634 (51.9%)	908 (51.8%)	
Collateral channel distribution	Ipsilateral only	3311 (47.2%)	896 (51.1%)	0.23
	Contralateral only	5988 (85.4%)	1478 (84.4%)	0.25
	Both of ipsilateral and contralateral	2452 (35.0%)	620 (35.4%)	0.75
CTO vessel diameter	Unmeasurable	33 (0.5%)	10 (0.6%)	0.65
	<2.5 mm	1614 (23.0%)	414 (23.6%)	
	≥2.5 mm and <3.0 mm	3178 (45.3%)	769 (43.9%)	
	≥3.0 mm and <3.5 mm	1818 (25.9%)	461 (26.3%)	
	≥3.5 mm	365 (5.2%)	98 (5.6%)	

CTO distal diameter	≥3.0 mm	242 (3.5%)	54 (3.1%)	0.65
	≥1.0 mm and <3.0 mm	5275 (75.3%)	1314 (75.0%)	
	<1.0 mm	1491 (21.3%)	384 (21.9%)	
CTO distal visibility	Good	4213 (60.1%)	1044 (59.6%)	0.47
	Fair	2718 (38.8%)	694 (39.6%)	
	Invisible	77 (1.1%)	14 (0.8%)	
CTO entry	Tapered/tunnel	4548 (64.9%)	1172 (66.9%)	0.14
	Blunt	1293 (18.5%)	289 (16.5%)	
	No stump	1167 (16.7%)	291 (16.6%)	
Calcification	Non	3482 (49.7%)	861 (49.1%)	0.95
	Mild	2086 (29.8%)	527 (30.1%)	
	Moderate	951 (13.6%)	236 (13.5%)	
	Severe	490 (7.0%)	128 (7.32%)	
Lesion bending		1480 (21.1%)	368 (21.0%)	0.92
Proximal tortuosity	Straight	3567 (50.9%)	911 (52.0%)	0.82
	Mild	2287 (32.6%)	556 (31.7%)	
	Moderate	971 (13.9%)	243 (13.9%)	
	Severe	183 (2.6%)	42 (2.4%)	
Lesion length	<20 mm	3158 (45.1%)	797 (45.5%)	0.82
	≥20 mm	3753 (53.6%)	928 (53.0%)	
	Unmeasurable	97 (1.4%)	27 (1.5%)	
Side branch at proximal cap		1885 (26.9%)	482 (27.5%)	0.61
Bifurcation at exit point		1310 (18.7%)	354 (20.2%)	0.15
Tandem CTO		217 (3.1%)	52 (3.0%)	0.78
Reattempt	Reattempted by the same operator	124 (1.8%)	33 (1.9%)	0.75
	Reattempted by another operator	1178 (16.8%)	289 (16.5%)	0.75
	Not reattempt	5706 (81.4%)	1430 (81.6%)	0.85

ISR CTO	947 (13.5%)	218 (12.4%)	0.24
AHA Segment-01 diseased	2401 (34.3%)	595 (34.0%)	0.81
AHA Segment-02 diseased	2532 (36.1%)	611 (34.9%)	0.33
AHA Segment-03 diseased	1506 (21.5%)	391 (22.3%)	0.45
AHA Segment-04 diseased	668 (9.5%)	173 (9.9%)	0.66
AHA Segment-06 diseased	2309 (32.9%)	583 (33.3%)	0.79
AHA Segment-07 diseased	2588 (36.9%)	666 (38.0%)	0.40
AHA Segment-08 diseased	248 (3.5%)	62 (3.5%)	1.00
AHA Segment-09 diseased	829 (11.8%)	219 (12.5%)	0.44
AHA Segment-10 diseased	123 (1.8%)	27 (1.5%)	0.54
AHA Segment-11 diseased	1182 (16.9%)	304 (17.4%)	0.63
AHA Segment-12 diseased	555 (7.9%)	145 (8.3%)	0.62
AHA Segment-13 diseased	2047 (29.2%)	527 (30.1%)	0.47
AHA Segment-14 diseased	389 (5.6%)	105 (6.0%)	0.47
AHA Segment-15 diseased	161 (2.2%)	44 (2.5%)	0.60
Diseased RCA	4938 (70.5%)	1220 (69.6%)	0.50
Diseased LAD	4308 (61.5%)	1099 (62.7%)	0.33
Diseased LCX	3290 (46.9%)	856 (48.9%)	0.15
Diseased LM-orifice	51 (0.7%)	10 (0.6%)	0.48
Diseased LM-body	93 (1.3%)	16 (0.9%)	0.16
Diseased LM-bifurcation	149 (2.1%)	38 (2.2%)	0.91
Diseased Graft	123 (1.8%)	25 (1.4%)	0.34

Values are presented as means±standard deviation or as numbers (percentages).

AHA, American Heart Association; CABG, coronary artery bypass grafting; CC, collateral channel; CCS, Canadian Cardiovascular Society; Cr, creatinine; CTO, chronic total occlusion; eGFR, estimated glomerular filtration rate; ISR, in-stent restenosis; J-CTO, Multicenter CTO Registry in Japan; LAD, left anterior descending artery; LCX, left circumflex artery; LM, left main coronary artery; LVEF, left ventricular ejection fraction; MI, myocardial infarction; NYHA, New York Heart Association; PCI, percutaneous coronary intervention; RCA, right coronary artery.
