# Supplementary material for: Machine Learning for Prediction of Technical Results of Percutaneous Coronary Intervention for Chronic Total Occlusion
Source: J Clin Med. 2023 May 9;12(10):3354. doi: 10.3390/jcm12103354 (PMC10218988; doi:10.3390/jcm12103354)
Supplement: Supplementary file 1 [file jcm-12-03354-s001.zip › suppleTableS4-JCM.pdf]

Supplemental Table S4. Patient characteristics in the training cohort

		**Overall procedures (n=7008)**	**Failed CTO-PCI (n=616)**	**Successful CTO-PCI (n=6392)**	***P* value**
Age, years		67.4±11.1	68.1±11.0	67.4±11.2	0.094
Female		1062 (15.2%)	104 (16.9%)	958 (15.0%)	0.21
Hypertension		5451 (77.8%)	504 (81.8%)	4947 (77.4%)	0.012
Hyperlipidemia		5592 (79.8%)	491 (79.7%)	5101 (79.8%)	0.96
Diabetes		3173 (45.3%)	308 (50.0%)	2865 (44.8%)	0.014
Smoking status	Never	2830 (40.4%)	252 (40.9%)	2578 (40.3%)	0.34
	Past	3021 (43.1%)	275 (44.6%)	2746 (43.0%)	
	Current	1157 (16.5%)	89 (14.4%)	1068 (16.7%)	
History of MI		3378 (48.2%)	311 (50.5%)	3067 (48.0%)	0.24
Prior CABG		494 (7.0%)	77 (12.5%)	417 (6.5%)	<0.001
Prior PCI		4780 (68.2%)	451 (73.2%)	4329 (67.7%)	0.005
Cerebrovascular disease		512 (7.3%)	48 (7.8%)	464 (7.3%)	0.63
Cr, mg/dL		1.23±1.49	1.52±2.11	1.20±1.42	<0.001
eGFR, mL/min/1.73 m^2		63.4±22.5	60.5±24.5	63.7±22.3	0.001
Hemodialysis		448 (6.4%)	76 (12.3%)	372 (5.8%)	<0.001
Chronic occlusive pulmonary disease		164 (2.3%)	23 (3.7%)	141 (2.2%)	0.017
Arteriosclerosis obliterans		834 (11.9%)	91 (14.8%)	743 (11.6%)	0.021
Malignancy		192 (2.7%)	18 (2.9%)	174 (2.7%)	0.77
EuroSCORE II		1.57±2.38	1.66±1.82	1.56±2.43	0.29
LVEF, %		54.3±13.0	53.6±12.8	54.4±13.0	0.15
NYHA class	Not heart failure	5528 (78.9%)	473 (76.8%)	5055 (79.1%)	0.23
	I	646 (9.2%)	64 (10.4%)	582 (9.1%)	
	II	617 (8.8%)	60 (9.7%)	557 (8.7%)	
	III	155 (2.2%)	10 (1.6%)	145 (2.3%)	

	IV	62 (0.9%)	9 (1.5%)	53 (0.8%)	
CCS class	Asymptomatic	3239 (46.2%)	291 (47.2%)	2948 (46.1%)	0.93
	I	1438 (20.5%)	123 (20.0%)	1315 (20.6%)	
	II	1992 (28.4%)	162 (26.3%)	1830 (28.6%)	
	III	250 (3.6%)	31 (5.0%)	219 (3.4%)	
	IV	89 (1.3%)	9 (1.5%)	80 (1.3%)	
ST-T wave abnormality on ECG		1688 (23.8%)	142 (23.1%)	1526 (23.9%)	0.65
Abnormal Q wave on ECG		1897 (27.1%)	165 (26.8%)	1732 (27.1%)	0.87
Stress Test	Not performed	6001 (85.6%)	519 (84.3%)	5482 (85.8%)	0.31
	Negative study	333 (4.8%)	39 (6.3%)	294 (4.6%)	0.054
	Positive study	632 (9.0%)	52 (8.4%)	580 (9.1%)	0.60
	Equivocal study	42 (0.6%)	6 (1.0%)	36 (0.6%)	0.21
Wall Motion in the perfusion territory of CTO	Normal	2780 (39.7%)	221 (35.9%)	2559 (40.0%)	0.21
	Hypokinesis	3896 (55.6%)	366 (59.4%)	3530 (55.2%)	
	Akinesis	316 (4.5%)	27 (4.4%)	289 (4.5%)	
	Dyskinesis	16 (0.2%)	2 (0.3%)	14 (0.2%)	
Viable CTO territory		6901 (98.5%)	602 (97.7%)	6299 (98.5%)	0.11
Diagnosis	Unstable angina pectoris	186 (2.7%)	16 (2.6%)	170 (2.7%)	0.93
	Silent myocardial ischemia	2624 (37.4%)	250 (40.6%)	2374 (37.1%)	0.092
	Stable angina pectoris	3056 (43.6%)	252 (40.9%)	2804 (43.9%)	0.16
	Old myocardial infarction	1075 (15.3%)	91 (14.8%)	984 (15.4%)	0.68
	Acute myocardial infarction	67 (1.0%)	7 (1.1%)	60 (0.9%)	0.63
Other than the operator's affiliated institution		2359 (33.7%)	209 (33.9%)	2150 (33.6%)	0.88
Grafted CTO vessel	CTO vessel grafted and graft failure (-)	87 (1.2%)	12 (1.9%)	75 (1.2%)	0.097
	CTO vessel grafted and graft failure (+)	311 (4.4%)	49 (8.0%)	262 (4.1%)	<0.001

	Not grafted CTO vessel	6610 (94.3%)	555 (90.1%)	6055 (94.7%)	<0.001
Initially planned strategy, Primary bidirectional approach		2045 (29.2%)	242 (39.3%)	1803 (28.2%)	<0.001
Number of diseased vessels	Single	3047 (43.5%)	247 (40.1%)	2800 (43.8%)	0.011
	Double	2239 (31.9%)	187 (30.4%)	2052 (32.1%)	
	Triple	1722 (24.6%)	182 (29.5%)	1540 (24.1%)	
Target CTO vessel	RCA	3499 (49.9%)	333 (54.1%)	3166 (49.5%)	0.032
	LAD	2267 (32.3%)	172 (27.9%)	2095 (32.8%)	0.014
	LCX	1210 (17.3%)	110 (17.9%)	1100 (17.2%)	0.68
	LM	29 (0.4%)	1 (0.2%)	28 (0.4%)	0.31
	Graft	3 (0.04%)	0 (0%)	3 (0.05%)	0.59
CTO location	Distal	736 (10.5%)	55 (8.9%)	681 (10.7%)	0.18
	Mid	2940 (42.0%)	252 (40.9%)	2688 (42.1%)	0.58
	Proximal	3050 (43.5%)	270 (43.8%)	2780 (43.5%)	0.87
	Ostium	282 (4.0%)	39 (6.3%)	243 (3.8%)	0.002
Collateral channel classification	CC0	387 (5.5%)	40 (6.5%)	347 (5.4%)	0.20
	CC1	2987 (42.6%)	276 (44.8%)	2711 (42.4%)	
	CC2	3634 (51.9%)	300 (48.7%)	3334 (52.2%)	
Collateral channel distribution	Ipsilateral only	3311 (47.2%)	280 (45.5%)	3031 (47.4%)	0.35
	Contralateral only	5988 (85.4%)	526 (85.4%)	5462 (85.5%)	0.97
	Both of ipsilateral and contralateral	2452 (35.0%)	207 (33.6%)	2245 (35.1%)	0.45
CTO vessel diameter	Unmeasurable	33 (0.5%)	8 (1.3%)	25 (0.4%)	<0.001
	<2.5 mm	1614 (23.0%)	173 (28.1%)	1441 (22.5%)	
	≥2.5 mm and <3.0 mm	3178 (45.3%)	235 (38.1%)	2943 (46.0%)	
	≥3.0 mm and <3.5 mm	1818 (25.9%)	162 (26.3%)	1656 (25.9%)	
	≥3.5 mm	365 (5.2%)	38 (6.2%)	327 (5.1%)	

CTO distal diameter	≥3.0 mm	242 (3.5%)	14 (2.3%)	228 (3.6%)	0.021
	≥1.0 mm and <3.0 mm	5275 (75.3%)	448 (72.7%)	4827 (75.5%)	
	<1.0 mm	1491 (21.3%)	154 (25.0%)	1337 (20.9%)	
CTO distal visibility	Good	4213 (60.1%)	318 (51.6%)	3895 (60.9%)	<0.001
	Fair	2718 (38.8%)	283 (45.9%)	2435 (38.1%)	
	Invisible	77 (1.1%)	15 (2.4%)	62 (1.0%)	
CTO entry	Tapered/tunnel	4548 (64.9%)	357 (58.0%)	4191 (65.6%)	<0.001
	Blunt	1293 (18.5%)	125 (20.3%)	1168 (18.3%)	
	No stump	1167 (16.7%)	134 (21.8%)	1033 (16.2%)	
Calcification	Non	3482 (49.7%)	251 (40.7%)	3231 (50.5%)	<0.001
	Mild	2086 (29.8%)	159 (25.8%)	1926 (30.1%)	
	Moderate	951 (13.6%)	106 (17.2%)	845 (13.2%)	
	Severe	490 (7.0%)	100 (16.2%)	390 (6.1%)	
Lesion bending		1480 (21.1%)	199 (32.3%)	1281 (20.0%)	<0.001
Proximal tortuosity	Straight	3567 (50.9%)	288 (46.8%)	3279 (51.3%)	<0.001
	Mild	2287 (32.6%)	192 (31.2%)	2095 (32.8%)	
	Moderate	971 (13.9%)	110 (17.9%)	861 (13.5%)	
	Severe	183 (2.6%)	26 (4.2%)	157 (2.5%)	
Lesion length	<20 mm	3158 (45.1%)	202 (32.8%)	2956 (46.2%)	<0.001
	≥20 mm	3753 (53.6%)	400 (64.9%)	3353 (52.5%)	
	Unmeasurable	97 (1.4%)	14 (2.3%)	83 (1.3%)	
Side branch at proximal cap		1885 (26.9%)	187 (30.4%)	1698 (26.6%)	0.043
Bifurcation at exit point		1310 (18.7%)	121 (19.6%)	1189 (18.6%)	0.53
Tandem CTO		217 (3.1%)	35 (5.7%)	182 (2.8%)	<0.001
Reattempt	Reattempted by the same operator	124 (1.8%)	20 (3.2%)	104 (1.6%)	0.004
	Reattempted by another	1178 (16.8%)	135 (21.9%)	1043 (16.3%)	<0.001

	operator				
	Not reattempt	5706 (81.4%)	461 (74.8%)	5245 (82.1%)	<0.001
ISR CTO		947 (13.5%)	69 (11.2%)	878 (13.7%)	0.079
AHA Segment-01 diseased		2401 (34.3%)	261 (42.4%)	2140 (33.5%)	<0.001
AHA Segment-02 diseased		2532 (36.1%)	229 (37.2%)	2303 (36.0%)	0.57
AHA Segment-03 diseased		1506 (21.5%)	133 (21.6%)	1373 (21.5%)	0.95
AHA Segment-04 diseased		668 (9.5%)	66 (10.7%)	602 (9.4%)	0.30
AHA Segment-06 diseased		2309 (32.9%)	202 (32.8%)	2107 (33.0%)	0.93
AHA Segment-07 diseased		2588 (36.9%)	221 (35.9%)	2367 (37.0%)	0.57
AHA Segment-08 diseased		248 (3.5%)	28 (4.5%)	220 (3.4%)	0.16
AHA Segment-09 diseased		829 (11.8%)	80 (13.0%)	749 (11.7%)	0.35
AHA Segment-10 diseased		123 (1.8%)	14 (2.3%)	109 (1.7%)	0.31
AHA Segment-11 diseased		1182 (16.9%)	135 (21.9%)	1047 (16.4%)	<0.001
AHA Segment-12 diseased		555 (7.9%)	42 (6.8%)	513 (8.0%)	0.29
AHA Segment-13 diseased		2047 (29.2%)	195 (31.7%)	1852 (29.0%)	0.16
AHA Segment-14 diseased		389 (5.6%)	47 (7.6%)	342 (5.4%)	0.018
AHA Segment-15 diseased		161 (2.2%)	13 (2.1%)	148 (2.3%)	0.75
Diseased RCA		4938 (70.5%)	469 (76.1%)	4469 (69.9%)	0.001
Diseased LAD		4308 (61.5%)	361 (58.6%)	3947 (61.7%)	0.13
Diseased LCX		3290 (46.9%)	321 (52.1%)	2969 (46.4%)	0.007
Diseased LM-orifice		51 (0.7%)	4 (0.6%)	47 (0.7%)	0.81
Diseased LM-body		93 (1.3%)	9 (1.5%)	84 (1.3%)	0.76
Diseased LM-bifurcation		149 (2.1%)	15 (2.4%)	134 (2.1%)	0.58
Diseased graft		123 (1.8%)	178 (2.9%)	105 (1.6%)	0.021

Values are presented as means±standard deviation or as numbers (percentages).

AHA, American Heart Association; CABG, coronary artery bypass grafting; CC, collateral channel; CCS, Canadian Cardiovascular Society; Cr, creatinine; CTO, chronic total occlusion; eGFR, estimated glomerular filtration rate; ISR, in-stent restenosis; J-CTO, Multicenter CTO Registry in Japan; LAD, left anterior descending artery; LCX, left circumflex

artery; LM, left main coronary artery; LVEF, left ventricular ejection fraction; MI, myocardial infarction; NYHA, New York Heart Association; PCI, percutaneous coronary intervention; RCA, right coronary artery.
