# Supplementary material for: Machine Learning for Prediction of Technical Results of Percutaneous Coronary Intervention for Chronic Total Occlusion
Source: J Clin Med. 2023 May 9;12(10):3354. doi: 10.3390/jcm12103354 (PMC10218988; doi:10.3390/jcm12103354)
Supplement: Supplementary file 1 [file jcm-12-03354-s001.zip › suppleTableS5-JCM.pdf]

Supplemental Table S5. Patient characteristics in the test cohort

		**Overall population (n=1752)**	**Failed CTO-PCI (n=154)**	**Successful CTO-PCI (n=1598)**	***P* value**
Age, years		67.3±10.8	66.6±10.2	67.4±10.9	0.43
Female		232 (13.2%)	20 (13.0%)	212 (13.3%)	0.92
Hypertension		1350 (77.1%)	122 (79.2%)	1228 (76.8%)	0.50
Hyperlipidemia		1412 (80.6%)	126 (81.8%)	1268 (80.5%)	0.69
Diabetes		781 (44.6%)	71 (46.1%)	710 (44.4%)	0.69
Smoking status	Never	651 (37.2%)	62 (40.3%)	589 (36.9%)	0.43
	Past	800 (45.7%)	71 (46.1%)	729 (45.6%)	
	Current	301 (17.2%)	21 (13.6%)	280 (17.5%)	
History of MI		8183 (46.4%)	75 (48.7%)	738 (46.2%)	0.55
Prior CABG		121 (6.9%)	17 (11.0%)	104 (6.5%)	0.034
Prior PCI		1205 (68.8%)	127 (82.5%)	1078 (67.5%)	<0.001
Cerebrovascular disease		132 (7.5%)	13 (8.4%)	119 (7.4%)	0.66
Cr, mg/dL		1.23±1.50	1.83±2.72	1.17±1.31	<0.001
eGFR, mL/min/1.73 m^2		63.5±22.8	59.1±26.1	63.9±22.4	0.012
Hemodialysis		137 (7.8%)	23 (14.9%)	114 (7.1%)	<0.001
Chronic occlusive pulmonary disease		50 (2.9%)	4 (2.6%)	46 (2.9%)	0.84
Arteriosclerosis obliterans		235 (13.4%)	29 (18.8%)	206 (12.9%)	0.039
Malignancy		53 (3.0%)	1 (0.6%)	52 (3.3%)	0.072
EuroSCORE II		1.50±1.44	1.62±1.49	1.49±1.44	0.27
LVEF, %		53.9±13.0	52.8±13.4	54.0±12.9	0.26
NYHA class	Not heart failure	1383 (78.9%)	121 (78.6%)	1262 (79.0%)	0.99
	I	155 (8.8%)	14 (9.1%)	141 (8.8%)	
	II	165 (9.4%)	15 (9.7%)	150 (9.4%)	
	III	36 (2.1%)	3 (1.9%)	33 (2.1%)	

	IV	13 (0.7%)	1 (0.6%)	12 (0.8%)	
CCS class	Asymptomatic	836 (47.7%)	78 (50.6%)	758 (47.4%)	0.27
	I	356 (20.3%)	37 (24.0%)	319 (20.0%)	
	II	471 (26.9%)	34 (22.1%)	437 (27.3%)	
	III	67 (3.8%)	5 (3.2%)	62 (3.9%)	
	IV	22 (1.3%)	0 (0%)	22 (1.4%)	
ST-T wave abnormality on ECG		426 (24.3%)	38 (24.7%)	388 (24.3%)	0.91
Abnormal Q wave on ECG		461 (26.3%)	45 (29.2%)	416 (26.0%)	0.39
Stress Test	Not performed	1529 (87.3%)	125 (81.2%)	1404 (87.9%)	0.017
	Negative study	76 (4.3%)	10 (6.5%)	66 (4.1%)	0.17
	Positive study	138 (7.9%)	17 (11.0%)	121 (7.6%)	0.13
	Equivocal study	9 (0.5%)	2 (1.3%)	7 (0.4%)	0.15
Wall Motion in the perfusion territory of CTO	Normal	678 (38.7%)	54 (35.1%)	624 (39.0%)	0.62
	Hypokinesis	1003 (57.2%)	95 (61.7%)	908 (56.8%)	
	Akinesis	66 (3.8%)	5 (3.2%)	61 (3.8%)	
	Dyskinesis	5 (0.3%)	0 (0%)	5 (0.3%)	
Viable CTO territory		1738 (99.2%)	151 (98.1%)	1587 (99.3%)	0.094
Diagnosis	Unstable angina pectoris	52 (3.0%)	3 (1.9%)	49 (3.1%)	0.43
	Silent myocardial ischemia	681 (38.9%)	60 (39.0%)	621 (38.9%)	0.98
	Stable angina pectoris	759 (43.3%)	64 (41.6%)	695 (43.5%)	0.64
	Old myocardial infarction	243 (13.9%)	27 (17.5%)	216 (13.5%)	0.17
	Acute myocardial infarction	17 (1.0%)	0 (0%)	17 (1.1%)	0.20
Other than the operator’s affiliated institution		587 (33.5%)	59 (38.3%)	528 (33.0%)	0.19
Grafted CTO vessel	CTO vessel grafted and graft failure (-)	26 (1.5%)	4 (2.6%)	22 (1.4%)	0.23
	CTO vessel grafted and graft failure (+)	71 (4.1%)	7 (4.5%)	64 (4.0%)	0.75

	Not grafted CTO vessel	1655 (94.5%)	143 (92.9%)	1512 (94.6%)	0.36
Initially planned strategy, Primary bidirectional approach		513 (29.3%)	69 (44.8%)	444 (27.8%)	<0.001
Number of diseased vessels	Single	745 (42.5%)	51 (33.1%)	694 (43.4%)	0.036
	Double	550 (31.4%)	53 (34.4%)	497 (31.1%)	
	Triple	457 (26.1%)	50 (32.5%)	407 (25.5%)	
Target CTO vessel	RCA	875 (49.9%)	92 (59.7%)	783 (49.0%)	0.011
	LAD	547 (31.2%)	31 (20.1%)	516 (32.3%)	0.002
	LCX	325 (18.6%)	30 (19.5%)	295 (18.5%)	0.76
	LM	5 (0.3%)	1 (0.6%)	4 (0.3%)	0.38
	Graft	0 (0%)	0 (0%)	0 (0%)	-
CTO location	Distal	201 (11.5%)	17 (11.0%)	184 (11.5%)	0.86
	Mid	717 (40.9%)	51 (33.1%)	666 (41.7%)	0.039
	Proximal	759 (43.3%)	76 (49.4%)	683 (42.7%)	0.11
	Ostium	75 (4.3%)	10 (6.5%)	65 (4.1%)	0.16
Collateral channel	CC0	98 (5.6%)	11 (7.1%)	87 (5.4%)	0.13
	CC1	746 (42.6%)	75 (48.7%)	671 (42.0%)	
	CC2	908 (51.8%)	68 (44.2%)	840 (52.6%)	
Collateral channel distribution	Ipsilateral only	896 (51.1%)	61 (39.6%)	795 (49.7%)	0.016
	Contralateral only	1478 (84.4%)	132 (85.7%)	1346 (84.2%)	0.63
	Both of ipsilateral and contralateral	620 (35.4%)	42 (27.3%)	578 (36.2%)	0.028
CTO vessel diameter	Unmeasurable	10 (0.6%)	2 (1.3%)	8 (0.5%)	0.28
	<2.5 mm	414 (23.6%)	36 (23.4%)	378 (23.7%)	
	≥2.5 mm and <3.0 mm	769 (43.9%)	60 (39.0%)	709 (44.4%)	
	≥3.0 mm and <3.5 mm	461 (26.3%)	43 (27.9%)	418 (26.21%)	
	≥3.5 mm	98 (5.6%)	13 (8.4%)	85 (5.3%)	

CTO distal diameter	≥3.0 mm	54 (3.1%)	5 (3.2%)	49 (3.1%)	0.89
	≥1.0 mm and <3.0 mm	1314 (75.0%)	113 (73.4%)	1201 (75.2%)	
	<1.0 mm	384 (21.9%)	36 (23.4%)	348 (21.8%)	
CTO distal visibility	Good	1044 (59.6%)	78 (50.6%)	966 (60.5%)	0.022
	Fair	694 (39.6%)	73 (47.4%)	621 (38.9%)	
	Invisible	14 (0.8%)	3 (1.9%)	11 (0.7%)	
CTO entry	Tapered/tunnel	1172 (66.9%)	83 (53.9%)	1089 (68.1%)	<0.001
	Blunt	289 (16.5%)	30 (19.5%)	259 (16.2%)	
	No stump	291 (16.6%)	41 (26.6%)	250 (15.6%)	
Calcification	Non	861 (49.1%)	57 (37.0%)	804 (50.3%)	<0.001
	Mild	527 (30.1%)	41 (26.6%)	486 (30.4%)	
	Moderate	236 (13.5%)	27 (17.5%)	209 (13.1%)	
	Severe	128 (7.32%)	29 (18.8%)	99 (6.2%)	
Lesion bending		368 (21.0%)	64 (41.65%)	304 (19.0%)	<0.001
Proximal tortuosity	Straight	911 (52.0%)	69 (44.8%)	842 (52.7%)	0.031
	Mild	556 (31.7%)	50 (32.5%)	506 (31.7%)	
	Moderate	243 (13.9%)	27 (17.5%)	216 (13.5%)	
	Severe	42 (2.4%)	8 (5.2%)	34 (2.1%)	
Lesion length	<20 mm	797 (45.5%)	38 (24.7%)	759 (47.5%)	<0.001
	≥20 mm	928 (53.0%)	114 (74.0%)	814 (50.9%)	
	Unmeasurable	27 (1.5%)	2 (1.3%)	25 (1.6%)	
Side branch at proximal cap		482 (27.5%)	48 (31.2%)	434 (27.2%)	0.29
Bifurcation at exit point		354 (20.2%)	36 (23.4%)	318 (19.9%)	0.30
Tandem CTO		52 (3.0%)	8 (5.2%)	44 (2.8%)	0.088
Reattempt	Reattempted by the same operator	33 (1.9%)	8 (5.2%)	25 (1.6%)	0.002
	Reattempted by another	289 (16.5%)	34 (22.1%)	255 (16.0%)	0.051

	operator				
	Not reattempt	1430 (81.6%)	112 (72.7%)	1318 (82.5%)	0.003
ISR CTO		218 (12.4%)	15 (9.7%)	203 (12.7%)	0.29
AHA Segment-01 diseased		595 (34.0%)	73 (47.4%)	522 (32.7%)	<0.001
AHA Segment-02 diseased		611 (34.9%)	60 (39.0%)	551 (34.5%)	0.27
AHA Segment-03 diseased		391 (22.3%)	42 (27.3%)	349 (21.8%)	0.12
AHA Segment-04 diseased		173 (9.9%)	18 (11.7%)	155 (9.7%)	0.43
AHA Segment-06 diseased		583 (33.3%)	45 (29.2%)	538 (33.7%)	0.26
AHA Segment-07 diseased		666 (38.0%)	55 (35.7%)	611 (38.2%)	0.54
AHA Segment-08 diseased		62 (3.5%)	8 (5.2%)	54 (3.4%)	0.24
AHA Segment-09 diseased		219 (12.5%)	24 (15.6%)	195 (12.2%)	0.23
AHA Segment-10 diseased		27 (1.5%)	4 (2.6%)	23 (1.4%)	0.27
AHA Segment-11 diseased		304 (17.4%)	36 (23.4%)	268 (16.8%)	0.039
AHA Segment-12 diseased		145 (8.3%)	12 (7.8%)	133 (8.3%)	0.82
AHA Segment-13 diseased		527 (30.1%)	52 (33.8%)	475 (29.7%)	0.30
AHA Segment-14 diseased		105 (6.0%)	7 (4.5%)	98 (6.1%)	0.43
AHA Segment-15 diseased		44 (2.5%)	4 (2.6%)	40 (2.5%)	0.94
Diseased RCA		1220 (69.6%)	128 (83.1%)	1092 (68.3%)	<0.001
Diseased LAD		1099 (62.7%)	93 (60.4%)	1006 (63.0%)	0.53
Diseased LCX		856 (48.9%)	82 (53.2%)	774 (48.4%)	0.25
Diseased LM-orifice		10 (0.6%)	2 (1.3%)	8 (0.5%)	0.21
Diseased LM-body		16 (0.9%)	3 (1.9%)	13 (0.8%)	0.16
Diseased LM-bifurcation		38 (2.2%)	5 (3.2%)	33(2.1%)	0.34
Diseased graft		25 (1.4%)	5 (3.2%)	20 (1.3%)	0.046

Values are presented as means±standard deviation or as numbers (percentages).

AHA, American Heart Association; CABG, coronary artery bypass grafting; CC, collateral channel; CCS, Canadian Cardiovascular Society; Cr, creatinine; CTO, chronic total occlusion; eGFR, estimated glomerular filtration rate; ISR, in-stent restenosis; J-CTO, Multicenter CTO Registry in Japan; LAD, left anterior descending artery; LCX, left circumflex

artery; LM, left main coronary artery; LVEF, left ventricular ejection fraction; MI, myocardial infarction; NYHA, New York Heart Association; PCI, percutaneous coronary intervention; RCA, right coronary artery.
